# Supplementary material for: New mitochondrial primers for metabarcoding of insects, designed and evaluated using in silico methods
Source: Mol Ecol Resour. 2018 Oct 16;19(1):90–104. doi: 10.1111/1755-0998.12942 (PMC7379581; doi:10.1111/1755-0998.12942)
Supplement: Supplementary file 1 [file MEN-19-90-s001.pdf]

New mitochondrial primers for metabarcoding of insects, designed and evaluated using *in silico* methods

Daniel Marquina. Anders F. Andersson & Fredrik Ronquist

Supplementary material: Figures

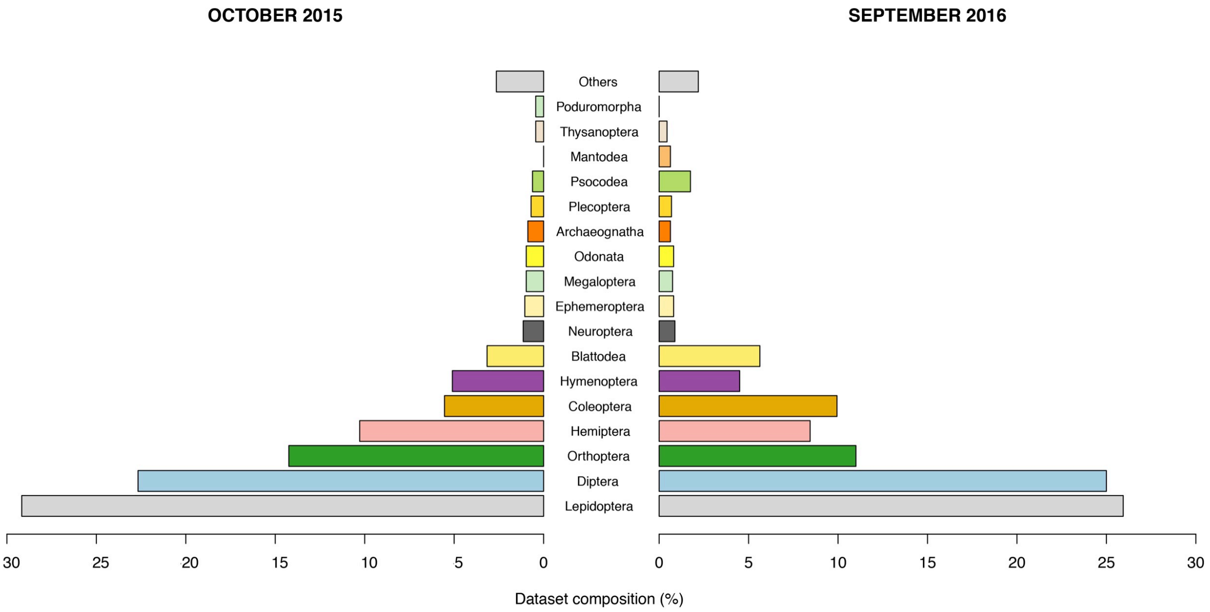

Fig. S1. Taxonomic composition of the mitochondrial genomes datasets used for design and evaluation of the primers.

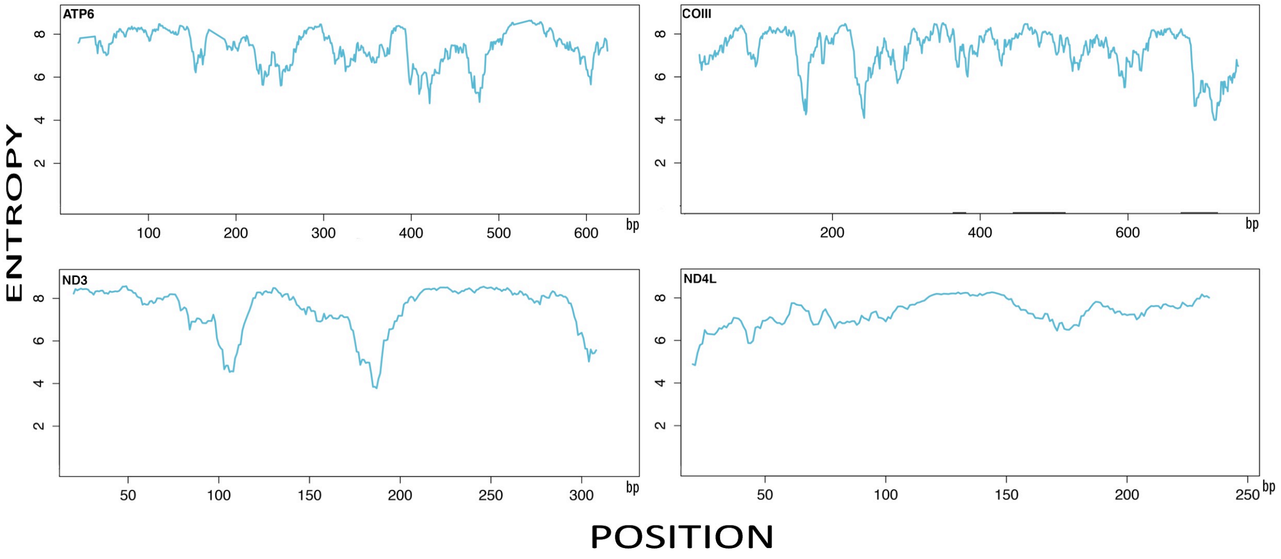

Fig. S2. Entropy as a function of potential primer site in the four genes for which we did not succeed in finding good PCR primers.

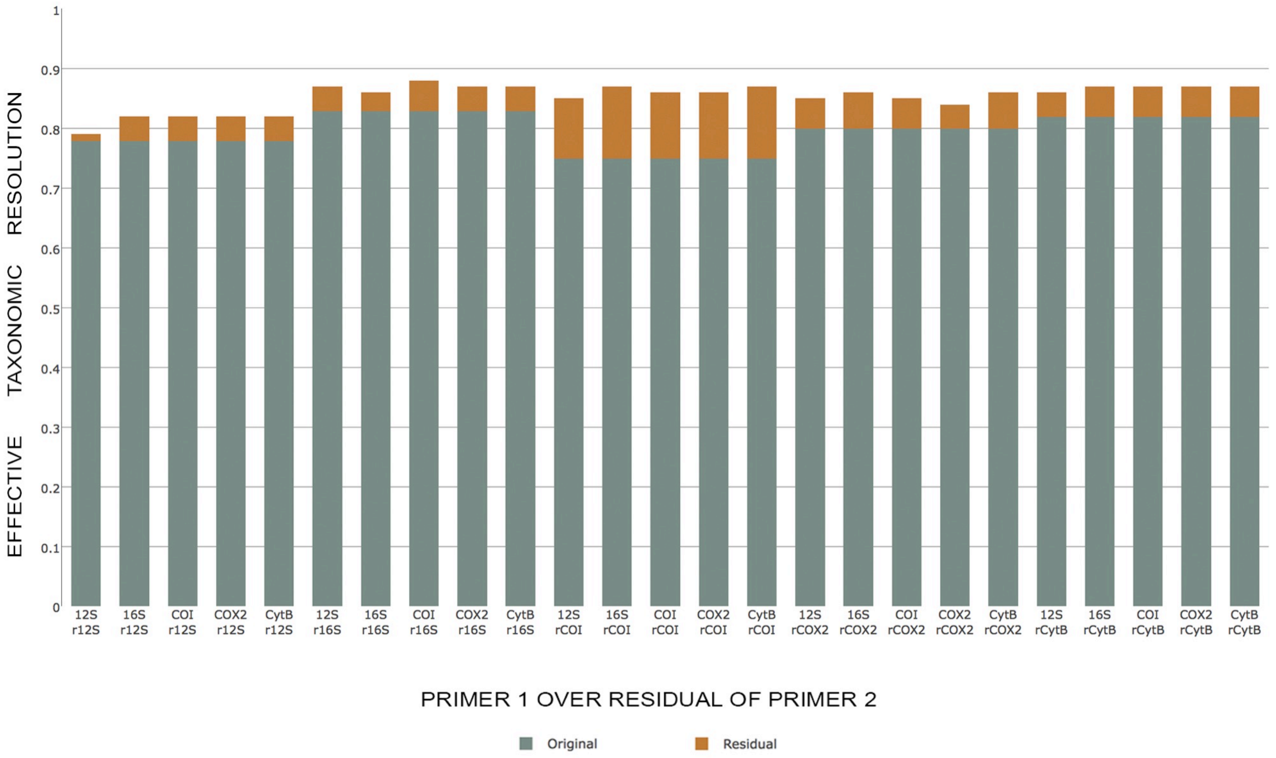

Fig. S3. Primer 2 over residual of primer 1 for all the markers with an original $ETR \geq 0.75$.
