# Supplementary material for: New mitochondrial primers for metabarcoding of insects, designed and evaluated using in silico methods
Source: Mol Ecol Resour. 2018 Oct 16;19(1):90–104. doi: 10.1111/1755-0998.12942 (PMC7379581; doi:10.1111/1755-0998.12942)
Supplement: Supplementary file 4 [file MEN-19-90-s004.pdf]

New mitochondrial primers for metabarcoding of insects, designed and evaluated using *in silico* methods

Daniel Marquina. Anders F. Andersson & Fredrik Ronquist

Supplementary material: Tables

Table S3. Primers designed using DEGEPRIME at a maximum degeneracy of 12-fold. Primer pairs Hex12SF1 – Hex12SR1 and Hex12SF2 – Hex12SR2 partially overlap with SR-J-14199 – SR-N-14594 published by Kambhampati & Smith (1994); primer Hex16SR2 partially overlaps with the reverse complement of Ins16S_9R published by Clarke *et al.* (2014); and primers HexCytBF1 and HexCytBF2 partially overlap with the reverse complement of REVCB2H published by Simmons & Weller (2001). The other primer pairs have not been discussed previously.

Marker	Primer Pair (name)	Primer Pair (sequence)	B_C	B_E'	ETR
**12S**	Hex12SF1 – Hex12SR1	HYTACTWTGTTACGACTT<br>HTAGRATTAGATACYCTA	0.81	0.88	0.71
	Hex12SF2 – Hex12SR2	ACTWTGTTACGACTTDTY<br>AGGATTAGATACCCTDBT	0.88	0.89	0.78
**16S**	Chiar16SF – Chiar16SR	TARTYCAACATCGRGGTC<br>CYGTRCDAAGGTAGCATA	0.93	0.89	0.83
	Hex16SF2 – Hex16SR2	GCTGTTATCCCYDARGTA<br>GTRCDAAGGTAGCATART	0.92	0.80	0.74
**COI**	HexCOIF1 – HexCOIR1	HATAATTTTYTTYATAGT<br>AARAATCARAATAARTGT	0.60	0.89	0.53
	HexCOIF2 – HexCOIR2	AATAAYATAAGHTTYTGA<br>AATTARAATRTADACTTC	0.57	0.86	0.49
	HexCOIF3 – HexCOIR3	CCHCGAATAAAYAAYATA<br>ATCARAATAARTGTTGRT	0.55	0.84	0.46
**COII**	HexCOX2F2 – HexCOX2R2	GATAYTGAAGHTAYGAAT<br>CCACARATTTCDGARCAT	0.52	0.89	0.46
**COIII**	HexCOX3F1 – HexCOX3R1	GGDATAATYYTATTTATT<br>DACAAARTGTCARTATCA	0.53	0.89	0.47
**CytB**	HexCytBF1 – HexCytBR1	DCAAATATCWTTYTGAGG<br>RAARTATCATTCDGGTTG	0.62	0.86	0.53
	HexCytBF2 – HexCytBR2	CAAATATCWTTYTGAGGD<br>ARTATCATTCDGGTTGRA	0.63	0.89	0.56
**ND5**	HexND5F2 – HexND5R2	ATCYTTWGAATAAAAHCC<br>GATTTDAARAARATTATT	0.55	0.89	0.49

Table S4. Primers designed using DEGEPRIME at a maximum degeneracy of 216-fold. Primer Hex12SR4 partially overlaps with SR-N-14594 published by Kambhampati & Smith (1994); primer pair HexCOIF4 – HexCOIR4 partially overlaps with BF2 – BR1 published by Elbrecht & Leese (2017) and ArF(1-5,10)–ArR(2,3,5,6,7,9) published by Gibson *et al.* 2014; primer HexCytBF3 partially overlaps with the reverse complement of REVCB2H published by Simmons & Weller (2001). The other primer pairs have not been discussed previously but they often represent more degenerate versions of the primer pairs found with DEGEPRIME at 12-fold degeneracy (Table S3).

Marker	Primer Pair (name)	Primer Pair (sequence)	B_C	B_E'	ETR
**12S**	Hex12SF3 – Hex12SR4	DYGAYGGGCDATDTGTRC<br>RAHHDGRATTAGATACYC	0.94	0.89	0.84
**16S**	Hex16SF3 – Hex12SR3	NTADYYCAACATCKRGGT<br>YYRYRCDAAGGTAGCRTA	0.96	0.89	0.85
**ATP6**	HexATP6F3 – HexATP6R4	HGCHAAYATRATYGCHGG<br>GHRAWHACATADGMTTGA	0.60	0.89	0.53
**COI**	HexCOIF4 – HexCOIR4	HCCHGAYATRGCHTTYCC<br>TATDGTRATDGCHCCNGC	0.83	0.90	0.75
**COII**	HexCOX2F3 – HexCOX2R3	GGNCRHCARTGRTAYTGA<br>RATYTCDGARCAYTGNCC	0.91	0.89	0.72
**COIII**	HexCOX3F2 – HexCOX3R2	DGCHACWGGDTTYCAYGG<br>ADGCDGCDGCTTCRAANC	0.67	0.88	0.59
**CytB**	HexCytBF3 – HexCytBR3	NCAAATRTCNTTHTGRGG<br>YCAYTCDGGYTKRATRTG	0.92	0.89	0.82
**ND1**	HexND1F3 – HexND1R3	YTCHGMAAARTCRAANGG<br>NTTYGAYTTTKCDGARGG	0.75	0.88	0.66
	HexND1F4 – HexND1R4	ATHARYTTATCRTANCGR<br>NTTYGAYTTTKCDGARGG	0.81	0.88	0.71
**ND3**	HexND3F2 – HexND3R2	YGARTGYGGDTTYRAYCC<br>TCATTCRTRRTADARNCC	0.64	0.89	0.57
**ND4**	HexND4F3 – HexND4R3	HGAHGAACAHARNCCATG<br>RGCNCATGTNGARGCHCC	0.74	0.89	0.66
	HexND4F4 – HexND4R4	HGGDGCYTCNACATGDGC<br>RGGNTAYCARCCDGARCG	0.75	0.89	0.67
**ND5**	HexND5F3 – HexND5R3	RTCYYTNGARTAAAAHCC<br>NGCHAAYTWTGARTWTGA	0.74	0.89	0.66
	HexND5F4 – HexND5R4	RTCWWAYTCAWARTTHGC<br>HTTRGTHCAYTCWTCDAC	0.65	0.89	0.58

Table S5. Primers designed using ECOPRIMERS.

Marker	Primer Pair (name)	Primer Pair (sequence)	B_C	B_E'	ETR
**16S**	Hex-eco1	ATAGAAACCAACCTGGCT	0.57	0.82	0.47
		TTACCTTAGGGATAACAG			
	Hex-eco2	ATAGAAACCAACCTGGCT	0.59	0.82	0.48
		TACCTTAGGGATAACAGC			
	Hex-eco3	GATAGAAACCAACCTGGC	0.59	0.82	0.48
		TACCTTAGGGATAACAGC			
	Hex-eco4	ATAGAAACCAACCTGGCT	0.64	0.80	0.51
		GACCTCGATGTTGGATTA			
	Hex-eco5	GATAGAAACCAACCTGGC	0.57	0.81	0.46
		TTACCTTAGGGATAACAG			

Table S6. Taxonomy annotation conflict between downloaded genomes from GenBank and BOLD database.

**Total species**				**1126**
	No hits			315
	Species =			686
	Species ≠			125
		Genus =		96
		Genus = ; sp.		7
		Genus ≠		29
		Family =		15
		Family ≠		14
			Order =	6
			Order ≠	8

Table S7. Proportion of unresolved species between the selected markers. Only species amplified by both markers were considered. Marker of reference in the rows for the upper triangle, and in the columns for the lower triangle.

	**12S**	**16S**	**COI**	**COII**	**CytB**	**ND1**	**ND4**	**ND5**
**12S**	-	0.71	0.71	0.68	0.76	0.73	0.73	0.73
**16S**	0.82	-	0.79	0.79	0.83	0.76	0.81	0.81
**COI**	0.75	0.73	-	0.81	0.76	0.79	0.79	0.78
**COII**	0.75	0.74	0.84	-	0.86	0.78	0.83	0.84
**CytB**	0.74	0.70	0.70	0.77	-	0.80	0.78	0.78
**ND1**	0.79	0.74	0.77	0.77	0.86	-	0.83	0.83
**ND4**	0.73	0.72	0.74	0.77	0.84	0.80	-	0.83
**ND5**	0.69	0.67	0.68	0.73	0.75	0.77	0.76	-

Table S8. Previously published primers.

Marker	Primer Pair (name)	Primer Pair (sequence)	B_C	B_E'	ETR	Reference
**12S**	SR-J-14199 – SR-N-14594	TACTATGTTACGACTTAT AAACTAGGATTAGATACCC	0.29	0.89	0.26	Kambhampati & Smith 1994
**16S**	Ins16S_1	TRRGACGAGAAGACCCTATA TCTTAATCCAACATCGAGGTC	0.06	-	-	Clarke *et al.* 2014
	Ins16S_1short	TRRGACGAGAAGACCCTATA ACGCTGTTATCCCTAAGGTA	0.17	0.84	0.14	Clarke *et al.* 2014
	Ins16S_9	GATAGAAACCAACCTGGCT AARTTACCTTAGGGATAACAGC	0.56	0.81	0.45	Clarke *et al.* 2014
	16SIns_F – Ins_R	RGACGAGAAGACCCTATARA ACGCTGTTATCCCTAARGTA	0.8	0.87	0.70	Elbrecth *et al.* 2016
	LR-J-12961 – LR-N-13398	TTTAATCCAACATCGAGG CGCCTGTTTAACAAAAACAT	0.05	-	-	Cognato & Vogler 2001 – Simon *et al.* 1994
	LR-J-13017 – LR-N-13398	TTACGCTGTTATCCCTAA CGCCTGTTTAACAAAAACAT	0.16	0.90	0.14	Kambhampati & Smith 1994 – Simon *et al*. 1994 –
**COI**	COIBF1 – COIBR1	ACWGGWTGRACWGTNTAYCC ARYATDGTRATDGCHCCDGC	0.68	0.89	0.61	Elbrecth & Leese 2017
	COIBF1 – COIBR2	ACWGGWTGRACWGTNTAYCC TCDGGRTGNCCRAARAAYCA	0.71	0.87	0.62	Elbrecth & Leese 2017
	COIBF2 – COIBR2	GCHCCHGAYATRGCHTTYCC TCDGGRTGNCCRAARAAYCA	0.87	0.90	0.78	Elbrecth & Leese 2017
	COIBF2 – COIBR1	GCHCCHGAYATRGCHTTYCC ARYATDGTRATDGCHCCDGC	0.82	0.89	0.73	Elbrecth & Leese 2017
	BE (ArF2 – ArR5)	CCIGAYATRGCITTYCCICG GTRATIGCICCIGCIARIAC	0.93	0.90	0.84	Gibson *et al.* 2014
	InsCOI	GTAAAGTAAGCTCGTGTATC TTATGCTATATTANCTATTGG	0.01	-	-	Willerslev *et al.* 2007
	dgLCO1490 – mlCOIintR	GGTCAACAAATCATAAAGAYA TYGG GGRGGRTASACSGTTCASCCSGT SCC	0	-	-	Meyer 2003 – Leray *et al.* 2013
	mlCOIintF – dgHCO2198	GGWACWGGWTGAACWGTWTA YCCYCC TAAACTTCAGGGTGACCAAARA AYCA	0.02	-	-	Leray *et al.* 2013 – Meyer 2003

	jgLCO1490–mlCOIintR	TITCIACIAAYCAYAARGAYATTGG GGRGGRTASACSGTTCASCCSGTSCC	0	-	-	Geller et al. 2013 - Leray et al. 2013
	mlCOIintF–jgHCO2198	GGWACWGGWTGAACWGTWTAYCCYCC TAIACYTCIGGRTGICCRAARAAYCA	0.4	0.90	0.36	Leray et al. 2013 – Leray et al. 2013
	ZBJ-ArctF1c – ZBJ-ArctR2c	AGATATTGGAACWTTATATTTTATTTTTGG WACTAATCAATTWCCAAATCCTCC	0.06	-	-	Zeale *et al*. 2011
	Ins3	TCCTGTTGGAACAGCAATAAT TCCTAAAGCTCCAAATGTTTCTTT	0	-	-	Thomsen *et al.* 2009
	LepF-EPT – long-univR	ATTCAACAAATCATAAAGATATTGG ACIITICACGCITTTRTTATRATTTTYTT	0	-	-	Hajibabaei *et al.* 2011
	LepF – Nancy	ATTCAACAAATCATAAAGATATTGG GAAGTTTATATTTTAATTTTACCAGG	0	-	-	Hajibabaei *et al.* 2011 – Silva-Brandão *et al.* 2008
	LepF1 – MLepF1-Rev	ATTCAACCAATCATAAAGATATTGG CACCWGATATAGCWTTTCCACG	0	-	-	Hajibabaei *et al.* 2011 – Brandon-Mong *et al.* 2015
	Uni-Minibar	GAAAATCATAATGAAGGCATGAGC GTACCAATATCYTTGTGATTAGTGGA	0	-	-	Meusnier *et al.* 2008
**CytB**	REVCB2H – REVCBJ	TGAGGACAAATATCATTTTGAGGW ACTGGTCGAGCTCCAATTCATGT	0.01	-	-	Simmons & Weller 2001

Table S9. Primers designed with DEGEPRIME over the residual of species not amplified by the selected best marker for 12S, 16S, COI, COII and CytB, respectively. When searching for these complementary primers, maximum degeneracy was set to 12 for 12S and 16S, and 216 for COI, COII and CytB.

Original marker	Targeted marker	Primer Pair (name)	Primer Pair (sequence)	B_C	B_E'	ETR
**12S**	12S	Hex12Sr12SF–Hex12Sr12SR	TACMHCTACTWTGTTACG<br>AAACYYAAAKAATTTGGC(2)	0.01	0.89	0.01
	16S	Hex16Sr12SF–Hex16Sr12SR	TADTYCAACATCKAGGTC<br>RACYGTRCAAAGGTAGCA	0.04	0.89	0.04
	COI	HexCOIr12SF–HexCOIr12SR(1)	HAAYCAYAARRAYATYGG<br>YCARTTHCCRAAHCCHCC	0.04	0.90	0.04
	COII	HexCOX2r12SF–HexCOX2r12SR	GGNCRHCAATGRTAYTGR<br>RATTTCHGMRCAYTGNCC	0.05	0.89	0.04
	CytB	HexCytBr12SF–HexCytBr12SR	AATRTCNTWYTGRGGDGC<br>WARRAARTAYCAYTCDGG	0.05	0.89	0.04
**16S**	12S	Hex12Sr16SF–Hex12Sr16SR	YGACGGGCRATDTGTACA(2)<br>WRACYAGGATTAGATACC	0.04	0.89	0.04
	16S	Hex16Sr126F–Hex16Sr16SR	YVCTGTTATCCCTAARGT(2)<br>GGTAGCATAATMADTWGT(2)	0.03	0.89	0.03
	COI	HexCOIr16SF–HexCOIr16SR	HCCHGAYATRGCHTTYCC<br>TATDGTRATDGCHCCNGC	0.05	0.90	0.05
	COII	HexCOX2r16SF–HexCOX2r16SR	GGNVVHCAATGATAYTGA<br>RATYTCHGMRCATTGNCC	0.05	0.89	0.04
	CytB	HexCytBr16SF–HexCytBr16SR	GNCAAATRTCNTTHTGRG<br>HARRAARTATCAYTCNGG	0.05	0.89	0.04
**COI**	12S	Hex12SrCOIF–Hex12SrCOIR	GACGGGCRATDTGTRCAT(2)<br>AHYAGGATTAGAKACCCT	0.11	0.89	0.10
	16S	Hex16SrCOIF–Hex16SrCOIR(1)	BTARTYCAACATCGAGGT<br>ACYGTRCDAAGGTAGCAT	0.14	0.89	0.12
	COI	HexCOIrCOIF–HexCOIrCOIR	NAAYCAYAARGAYATYGG<br>YCARTTHCCRAAHCCHCC	0.12	0.90	0.11
	COII	HexCOX2rCOIF–HexCOX2rCOIR	GGNCRHCAATGRTWYTGA<br>RATYTCDGARCAYTGNCC	0.12	0.89	0.11
	CytB	HexCytBrCOIF–HexCytBrCOIR	RATRTCHTTHTGRGGDGC<br>YCAYTCDGGYTKRATRTG	0.14	0.89	0.12
**COII**	12S	Hex12SrCOX2F–Hex12SrCOX2R	GACGGGCRATDTGTRCAT(2)<br>ARACBRGGATTAGATACC	0.06	0.89	0.05

	16S	Hex16SrCOX2F–Hex16SrCOX2R	YTARTYCAACATCGAGGT<br>ACYGTRCDAAGGTAGCAT	0.07	0.89	0.06
	COI	HexCOIrCOX2F–HexCOIrCOX2R	WGGDGGDTTYGGDAAYTG(2)<br>YATWGTRATDGCHCCDGC	0.06	0.90	0.05
	COII	HexCOX2rCOX2F–HexCOX2rCOX2R	HGGNMAYCAATGATWYTG<br>RATTTCHGMRCAYTGNCC	0.05	0.89	0.04
	CytB	HexCytBrCOX2F–HexCytBrCOX2R	GNCARATRTCHTTYTGRG<br>YCAYTCDGGYTKRATRTG	0.07	0.89	0.06
**CytB**	12S	Hex12SrCytBF–Hex12SrCytBR	GACGGGCRATDTGTRCAT(2)<br>CYAGGATTAGAKACCCTR	0.05	0.89	0.04
	16S	Hex16SrCytBF–Hex16SrCytBR	TARTYCAACATCGAGGTC<br>CYGTRCDAAGGTAGCATA	0.06	0.89	0.05
	COI	HexCOIrCytBF–HexCOIrCytBR	HCCHGAYATRGCHTTYCC<br>TATDGTRATDGCHCCNGC	0.06	0.90	0.05
	COII	HexCOX2rCytBF–HexCOX2rCytBR	GGNCRHCAATGRTAYTGR<br>RATYTCHGARCAYTGNCC	0.06	0.89	0.05
	CytB	HexCytBrCytBF–HexCytBrCytBR	RGGDGCHACWGTHATYAC<br>ARRAARTAYCAYTCHGGY	0.06	0.89	0.05

(1) indicates when the complementary amplicon does not overlap with the original amplicon for the gene (column 3) ; (2) indicates when the amplicon does overlap with the original amplicon for the original marker but the primer does not (column 4).

Table S10. Total effective taxonomic resolution (ETR_T) of each pairwise combination of selected primers (above diagonal) and redundant ETR (ETR_R) (below diagonal).

Primer Pair II<br>Primer Pair I	12S	16S	COI	COII	CytB
**12S**	-	0.85	0.88	0.85	0.85
**16S**	0.76	-	0.88	0.84	0.84
**COI**	0.72	0.71	-	0.89	0.83
**COII**	0.75	0.74	0.68	-	0.84
**CYTB**	0.76	0.75	0.68	0.73	-

Table S11. Uniquely contributed effective taxonomic resolution (ETR_U) for the two primers of each pairwise combination.

Primer Pair II<br>Primer Pair I	12S	16S	COI	COII	CytB
**12S**	-	0.05	0.12	0.06	0.05
**16S**	0.04	-	0.12	0.06	0.05
**COI**	0.04	0.05	-	0.06	0.05
**COII**	0.04	0.04	0.15	-	0.05
**CYTB**	0.04	0.04	0.12	0.06	-

References

Brandon–Mong GJ, Gan HM, Sing KW *et al.* (2015) DNA Metabarcoding of Insects and Allies: an Evaluation of Primers and Pipelines. *Bulletin of Entomological Research*, **105**, 717–727.

Clarke LJ, Soubrier J, Weyrich LS, Cooper A (2014) Environmental metabarcodes for insects: *in silico* PCR reveals potential for taxonomic bias. *Molecular Ecology Resources*, **14**, 1160–1170.

Vogler AP, Cognato AI (2001) Exploring Data Interaction and Nucleotide Alignment in a Multiple Gene Analysis of Ips (Coleoptera: Scolytinae). *Systematic Biology*, **50**, 758–780.

Elbrecht V, Taberlet P, Dejean T *et al,* (2016) Testing the potential of a ribosomal 16S marker for DNA metabarcoding of insects. *PeerJ*, **4**, e1966–12.

Elbrecht V, Leese F (2017) Validation and Development of COI Metabarcoding Primers for Freshwater Macroinvertebrate Bioassessment. *Frontiers in Environmental Science*, **5**, 314–11,

Gibson JF, Shokralla S, Porter TM *et al.* (2014) Simultaneous assessment of the macrobiome and microbiome in a bulk sample of tropical arthropods through DNA metasystematics. *Proceedings of the National Academy of Sciences*, **111**, 8007–8012.

Hajibabaei M, Shokralla S, Zhou X, Singer GAC, Baird DJ (2011) Environmental Barcoding: A Next–Generation Sequencing Approach for Biomonitoring Applications Using River Benthos. *PLoS ONE*, **6**, e17497–7.

Kambhampati S, Smith PT (1994) PCR primers for the amplification of four insect mitochondrial gene fragments. *Insect Molecular Biology*, **4**, 233–236.

Leray M, Yang JJ, Meyer CP *et al.* (2013) A new versatile primer set targeting a short fragment of the mitochondrial COI region for metabarcoding metazoan diversity: application for characterizing coral reef fish gut contents. *Frontiers in Zoology*, **10**, 34.

Meusnier I, Singer GAC, Landry JF *et al.* (2008) A universal DNA mini–barcode for biodiversity analysis. *BMC Genomics*, **9**, 214–4.

Meyer CP (2003) Molecular systematics of cowries (Gastropoda: Cypraeidae) and diversification patterns in the tropics. *Biological Journal of the Linnean Society*, **79**, 401–479.

Silva–Brandão KL, Wahlberg N, Francini RB *et al.* (2008) Phylogenetic relationships of butterflies of the tribe Acraeini (Lepidoptera. Nymphalidae. Heliconiinae) and the evolution of host plant use. *Molecular Phylogenetics and Evolution*, **46**, 515–531.

Simon C, Frati F, Beckenbach A *et al.* (2005) Evolution. weighting. and phylogenetic utility of mitochondrial gene sequences and a compilation of conserved polymerase chain reaction primers. *Annals of the Entomological Society of America*, **87**, 651–701.

Simmons RB, Weller SJ (2001) Utility and Evolution of Cytochrome b in Insects. *Molecular Phylogenetics and Evolution*, **20**, 196–210.

Thomsen PF, Kielgast J, Iversen LL et al. (2011) Monitoring endangered freshwater biodiversity using environmental DNA. *Molecular Ecology*, **21**, 2565–2573.

Willerslev E, Cappellini E, Boomsma W et al. (2007) Ancient biomolecules from deep ice cores reveal a forested southern Greenland. *Science*, **317**, 111–114.

Zeale MR, Butlin RK, Barker GLA, Lees DC, Jones G (2011) Taxon–specific PCR for DNA barcoding arthropod prey in bat faeces. *Molecular Ecology Resources*, **11**, 236–244.
